# Diagnostic contribution of multi-frequency vibrometry to detection of peripheral neuropathy in type 1 diabetes mellitus compared with nerve conduction studies

**DOI:** 10.1371/journal.pone.0296661

**Published:** 2024-01-10

**Authors:** Linnéa Ekman, Lars B. Dahlin, Gert S. Andersson, Eero Lindholm

**Affiliations:** 1 Department of Translational Medicine, Hand Surgery, Lund University, Malmö, Sweden; 2 Department of Hand Surgery, Skåne University Hospital, Malmö, Sweden; 3 Department of Biomedical and Clinical Sciences, Linköping University, Linköping, Sweden; 4 Department of Clinical Sciences, Clinical Neurophysiology, Lund University, Malmö, Sweden; 5 Department of Clinical Sciences, Endocrinology, Lund University, Malmö, Sweden; Iuliu Hațieganu University of Medicine and Pharmacy: Universitatea de Medicina si Farmacie Iuliu Hatieganu, ROMANIA

## Abstract

**Aim:**

The aim was to assess the use of multi-frequency vibrometry (MFV) in detecting diabetic peripheral neuropathy (DPN) in type 1 diabetes in comparison to nerve conduction studies (NCS) and neurothesiometer (NT). Our objectives were to examine how VPTs correlated with NCS parameters, evaluate the efficacy of MFV in distinguishing DPN as well as to investigate whether MFV procedure could be based on fewer frequencies.

**Methods:**

Adults with type 1 diabetes with previous MFV examinations were recruited at Skåne University Hospital in Malmö, Sweden, between 2018 and 2020. Participants were examined regarding nerve function in the lower limbs through MFV, NT and NCS.

**Results:**

A total of 66 participants (28 women and 38 men) with a median age of 50 (39 to 64) years were included in the study. Through NCS assessment, 33 participants (50%) were diagnosed with DPN. We found negative correlations between VPTs and all NCS parameters, where the strongest correlation was found between sural nerve amplitude and the 125 Hz frequency of MFV. A combination of four frequencies, two low (4 and 8 Hz) and two high (125 and 250 Hz), showed the highest classification efficacy (AUC 0.83, 95% CI 0.73–0.93).

**Conclusion:**

We conclude that a strong correlation exists between the sural nerve amplitude and the VPTs at 125 Hz and that VPT testing with MFV can be focused on only four frequencies instead of seven, thus shortening test time, to distinguish DPN in the lower limb.

## Introduction

Neuropathy is a common and serious complication to diabetes mellitus associated with devastating consequences such as chronic pain, diabetic foot ulceration, and lower limb amputations [1]. The reported prevalence numbers for diabetic peripheral neuropathy (DPN) vary depending on the population studied, but it is estimated to occur in up to 50% of all patients with diabetes [2]. The definition of DPN according to the American Diabetes Association is “presence of signs and/or symptoms of peripheral nerve dysfunction in people with diabetes after the exclusion of other causes” [3]. Although nerve conduction studies (NCS) are a validated and reliable method widely regarded as the gold standard for assessing large fiber neuropathy [4], it is rarely used in the context of evaluating prevalence of DPN. This is primarily due to the time-consuming and expensive examinations which makes it unsuitable for screening purposes [5]. Another limitation with NCS is the discomfort or even pain it may cause to patients [6]. Consequently, diagnosis of DPN predominantly relies on clinical evaluation methods such as assessment with tuning fork or a single monofilament. These methods are somewhat hampered by low sensitivity (21–41%) for detection of DPN in patients without foot ulcers [7, 8]. Alternative methods that are rapid and simple but still reliable are needed, where also early signs of neuropathy can be detected, in order to initiate treatment and preventive procedures.

Assessment of vibration perception thresholds (VPTs) with multi-frequency vibrometry (MFV) is a novel method that could be easily accessible and thus useful in a clinical setting. Numerous studies have demonstrated impaired VPTs across various types of neuropathies [9–12], and this impairment has shown to be associated with the risk of developing diabetic foot ulcers [13, 14]. Furthermore, a previous study showed that levels of VPTs can be improved through enhanced metabolic control in patients with type 1 diabetes mellitus [15]. While assessment of VPTs is already part of clinical practice, the conventional method involves the use of e.g., a neurothesiometer (NT) which is a hand-held device vibrating in a single frequency. In the present study, we assessed VPTs using both NT and MFV.

For MFV, a lower limb assessment involves VPT testing at seven frequencies (4, 8, 16, 32, 64, 125 and 250 Hz) at the first and fifth metatarsal heads (MTI and MTV) on the sole of the foot. However, this comprehensive examination, covering both feet, takes approximately 30 minutes to complete. To enhance its practicality, especially in the primary and secondary care testing time needs to be reduced. It is also unknown whether the VPTs measured with MFV correlate with electrophysiological variables of the NCS. Our overall aim with the study was to investigate the diagnostic contribution of MFV to detection of DPN in type 1 diabetes mellitus in comparison to NCS and the neurothesiometer (NT). Our first objective was to see how VPTs, measured by both MFV and NT, correlated with NCS parameters. The second objective was to investigate whether VPT testing with MFV could be based on fewer frequencies; thus, shortening the test procedure, as well as to evaluate its efficacy to distinguish DPN.

## Methods and materials

### Ethical approval

The regional ethical review board at Lund University approved the study (ethical permission 386/2007, 2015/683 and 2017/386). The study was conducted in accordance with the Declaration of Helsinki and written informed consent was obtained from all participants.

### Study design and population

In this cross-sectional study, adults with type 1 diabetes mellitus were recruited at the Department of Endocrinology at Skåne University Hospital in Malmö, Sweden between February 2018, and March 2020. Recruitment was based on previously recorded VPTs covering different grade of impairment from normal to severely impaired VPTs. In addition to prior MFV test, inclusion criteria were age above 18 years and type 1 diabetes mellitus. Exclusion criteria were persons with any other known condition causing neuropathy (e.g., vitamin B12 deficiency, hypothyroidism, previous treatment with chemotherapy and alcoholism). Participants of the study were investigated regarding nerve function in the lower limbs using MFV, NT and NCS. The examinations covered both legs, except in one case where the participant arrived late for NCS, and limited assessment time only allowed for one leg to be examined. All participants underwent all three examinations during the same day, except for one participant who had the NCS conducted nine days later.

### Nerve conduction studies

Nerve conduction studies were performed by a single biomedical scientist at the Department of Clinical Neurophysiology, Skåne University Hospital, Malmö, Sweden according to a standardized procedure. On stimulation of the tibial and peroneal nerves, motor conduction velocities and response amplitudes were recorded, while sensory conduction velocity and response amplitude were recorded from the sural nerve.

An experienced neurophysiologist (GA), blinded to information about the patient characteristics or results of the other nerve function tests, assessed the results of the NCS. The results were graded according to clinical routine as either healthy, borderline, or pathological. Borderline was further divided into probably healthy and probably pathological. If all values were normal, the results were regarded as healthy. If one or two nerves displayed values outside the reference values, the results were classified as borderline (one nerve: probably healthy, two nerves: probably pathological). If three or more nerves were affected, the results were regarded as pathological.

### Vibration perception thresholds

Vibration perception thresholds were examined through MFV using the VibroSense Meter I Device (VibroSense Dynamics AB, Malmö, Sweden). The examination was performed in an isolated and quiet room where the participant was comfortably seated and provided with hearing protection. VPTs were examined at MTI and MTV in the sole of both right and left foot. The investigated area was excited with a vibrating probe, 4 mm in diameter, carrying out vibrations in seven different frequencies (4, 8, 16, 32, 64, 125 and 250 Hz). Contact force of the probe as well as room and skin temperature was measured and controlled to be within limits according to the requirements of ISO 13091–1 [16]. The participant was instructed to press and hold a button when registering vibrations as well as to release it when no vibrations remained. A more detailed description of the VibroSense Meter and the examination procedure has been presented previously [17].

Vibration perception thresholds were also assessed with the Horwell Neurothesiometer (NT; Scientific Laboratory Supplies, Nottingham, U.K). Assessment was performed in a sitting position with the foot rested against the floor. The NT is a hand-held device, and the probe was applied perpendicularly and gently pressed against the bony prominent of the MTI. The vibratory output, with the preset frequency of 50 Hz, was gradually increased from zero and up until the participant reported that they could feel the vibration. Thresholds were measured twice at each foot and a mean of the two measurements was calculated. Assessment with both MFV and NT was performed blinded to the results from the NCS by either one of two examiners (LE and EL) at the Department of Endocrinology.

### Statistical analyses

The participants were dichotomized into two groups based on the NCS assessment: no DPN (including healthy and probably healthy) and DPN (including pathological and probably pathological). Data are presented as median (25^th^ to 75^th^ percentile) or numbers (%). Mann-Whitney U-test (two-tailed) or Chi-squared test were used for group comparisons and correlations were analyzed by two-tailed Spearman Rank Correlation test. In order to evaluate the number of frequencies needed in the test procedure, Z-scores based on age- and gender-matched controls [17] were calculated for each frequency and then combined into four models as follows: all seven frequencies, the two lowest frequencies (4 and 8 Hz), the two highest frequencies (125 and 250 Hz) or a combination of the two lowest and the two highest frequencies (4, 8, 125 and 250 Hz), of both MTI and MTV and from both feet. Receiver-operating characteristics (ROC) analyses were performed with DPN status as state variable and the combined Z scores as test variables. P-values <0.05 were considered statistically significant. Statistical analyses were performed using SPSS (IBM SPSS Statistics version 27).

## Results

### Characteristics and DPN assessment

A total of 67 participants with type 1 diabetes mellitus were recruited to the study. One participant discontinued the NCS due to discomfort and was thus not included for further analyses. Hence, the studied group consisted of 66 participants (28 women and 38 men) with a median age of 50 (39 to 64) years (Table 1). According to the NCS assessment, 27 participants had normal NCS results and 32 were defined as pathological. Six participants were defined as borderline/probably healthy and one participant as borderline/probably pathological. Thus, the two groups consisted of 33 participants each when dichotomized into DPN and no DPN. The characteristics of the participants, for the entire group as well as for those with and without DPN separately, are presented in Table 1. Participants with DPN had significantly longer diabetes duration (p<0.001) and higher HbA1c values (p = 0.042) than participants with no DPN. The group with DPN had higher VPTs, regarding both NT (p = 3.9*10^−9^) and MFV (125 Hz frequency at MTI and MTV, p = 9.5*10^−12^ and p = 4.6*10^−10^; all remaining frequencies p<0.001), and lower sural nerve amplitude (p = 1.4*10^−17^) compared to the group with no DPN (Table 1).

**Table 1:** Clinical characteristics and examinations for participants with type 1 diabetes, with and without diabetic peripheral neuropathy (DPN).

Characteristics	All participants N = 66	No DPN N = 33	DPN n = 33
AGE [YEARS]	50 [39–64]	47 [35–60]	55 [40–66]
FEMALE	28 (42)	16 (49)	12 (36)
LENGTH [CM]	173.0 [166.8–182.3]	173.0 [166.0–181.0]	174.0 [167.5–186.0]
WEIGHT [KG]	73.0 [65.8–85.00]	73.0 [64.0–83.0]	74.0 [67.5–88.5]
DIABETES DURATION [YEARS]	27.7 [16.7–37.0]	20.1 [8.9–28.5]	**33.7 [26.7–44.8]**
HBA1C [MMOL/MOL]	56.0 [51.5–65.0]	54.0 [49.0–62.5]	**62.0 [54.0–65.5]**
NEUROTHESIOMETER [V]	20 [12–30]	15 [8–20]	**25 [22–40]**
SURAL NERVE AMPLITUDE [μV]	4.0 [0–8]	7.5 [5–12]	**0.0 [0–3]**
MTI 125 HZ [DB]	138.8 [127.6–146.1]	129.2 [117.8–139.3]	**145.0 [137.9–151.7]**
MTV 125 HZ [DB]	138.7 [115.3–144.1]	126.4 [117.1–139.4]	**142.8 [137.6–147.5]**

Data are presented as median [25^th^-75^th^ percentiles] or numbers (%). Significant values (p<0.05) between DPN and no DPN are highlighted in bold (Mann-Whitney U test).

DPN: diabetic peripheral neuropathy. MTI: first metatarsal head. MTV: fifth metatarsal head.

### Correlations between nerve conduction studies and vibration perception thresholds

**Fig 1:**
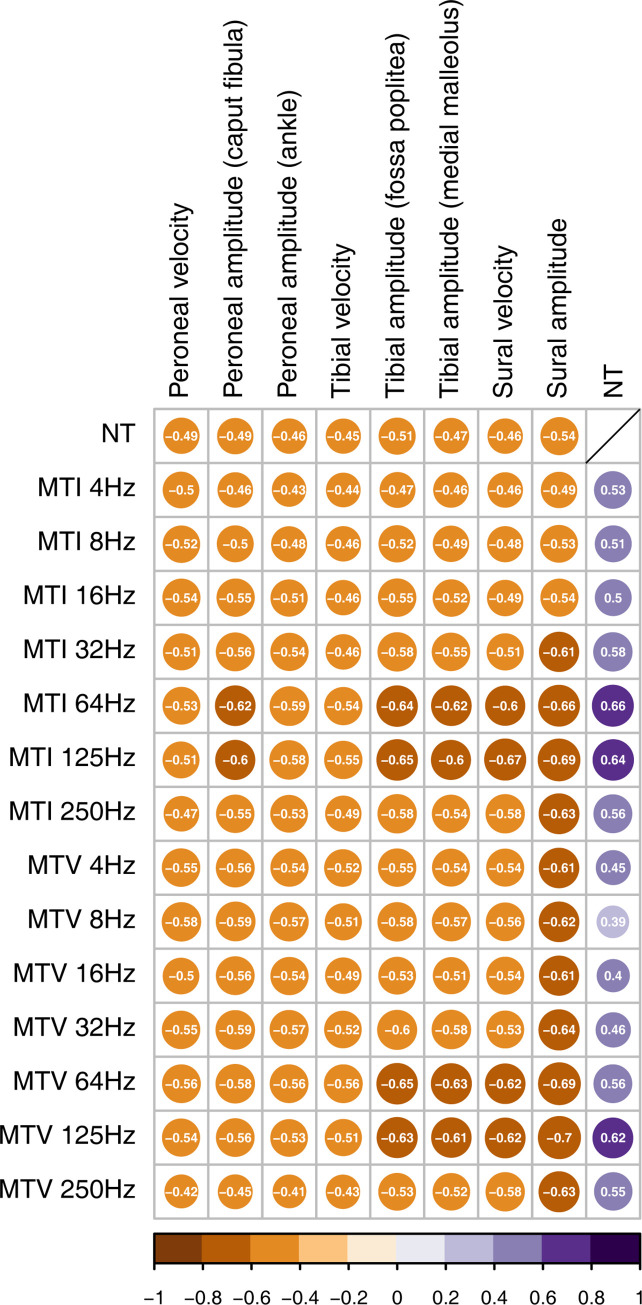
Heatmap of correlations between NCS parameters and VPTs. Correlation heatmap reporting Spearman correlation coefficients between nerve conduction studies (NCS) parameters and vibration perception thresholds (VPTs) measured with neurothesiometer (NT) or multi-frequency vibrometry (MFV) at first or fifth metatarsal head (MTI and MTV). Purple and brown color represent positive and negative correlation coefficients, according to the color legend. The darker the color and the more filled out the shape, the stronger the correlation. All correlations are significant, p<0.0001. Data based on 132 observations.

Vibration perception thresholds correlated negatively with all parameters from the NCS, whereas the NT correlated positively with all the VPTs from MFV (p<0.0001 for all; Fig 1). The strongest correlation was found between sural nerve amplitude and the 125 Hz frequency at MTV (r_s_ = -0.715, p = 1.7*10^−21^; Fig 1).

### Combinations of frequencies in test procedure

**Fig 2:**
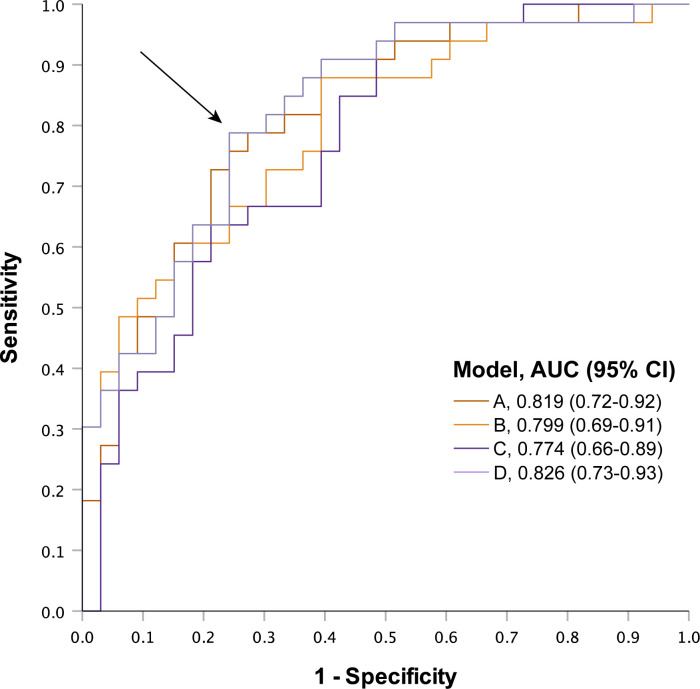
ROC curves of DPN classification by four different models of MFV. Receiver-operating characteristics (ROC) analyses with diabetic peripheral neuropathy (DPN) status as state variable and four models of combined frequencies from multi-frequency vibrometry (MFV) as test variables. Models consisted of A: all frequencies, B: two highest frequencies, C: two lowest frequencies or D: two lowest and two highest frequencies. Arrow marks cut-off point for model D based on maximum Youden’s index. AUC: area under curve, CI: confidence interval.

Out of the four models tested, the one containing both low and high frequencies (4, 8, 125 and 250 Hz) demonstrated the highest classification efficacy in determining whether participants had DPN or not (AUC 0.83, 95% CI 0.73–0.93; Fig 2). A cut-off value of total Z score = 0.499 showed a sensitivity of 79% and specificity of 76% (maximal Youden’s index; arrow in Fig 2).

## Discussion

The present study demonstrates that VPT assessment with MFV can be based on fewer frequencies than the original seven, and that the combination of two low as well as two high frequencies exhibited the highest classification efficacy in detecting DPN. We also found significant negative correlations between all MFV and NCS parameters, with the strongest observed correlation between sural nerve amplitude and the 125 Hz frequency measured at MTV. The generally stronger correlations observed with the sural nerve amplitude were anticipated, given that sensory NCS are more sensitive than motor NCS in terms of detecting axonal loss. Measures of sural nerve amplitude have also shown to be highly correlated to the morphological severity of DPN, as determined by nerve biopsy [18]. It was however unexpected that the strongest correlation was found for the 125 Hz frequency measured at MTV. Despite the widespread use of this frequency in clinical practice, such as with the 128 Hz tuning fork, it does not align with the ideal sensitivity ranges of either the Meissner or Pacinian corpuscles, which are the mechanoreceptors responsible for detecting vibration [19, 20]. Additionally, no differences could be found between VPTs measured at MTI and MTV among healthy adults [17]. Nevertheless, in a previous study, VPTs at 125 Hz was found to be impaired in patients with short-term type 1 diabetes mellitus without DPN, when compared to healthy controls [13]. In the same study, diabetic foot ulcers seemed to be associated with VPT measures at the MTV, although for the lower frequencies of 4 and 8 Hz. A supporting factor for the MTV’s significance is the higher density of cutaneous afferents distributed on the lateral part of the foot [21]. In addition to the previously mentioned strong correlation, most of the remaining correlations were moderate and sometimes even weak. Although proposed to be used for the same purpose, it is important to remember that MFV and NCS assess peripheral nerve function in two distinctively different ways. Whereas the NCS is considered fully objective and can be performed passively, the MFV requires active participation and cooperation from the examined patient. Thereto, MFV assesses the function of both mechanoreceptors in the skin and the afferent nerves, while NCS measures the function of a specific portion of the peripheral nerve, i.e., myelinated nerve fibers. Considering the different modalities and requirements involved, it is reasonable to expect that the methods are not fully comparable in terms of correlation. Moreover, our finding stands in contrast to a previous study, where MFV and NCS were compared in patients with carpal tunnel syndrome and no correlations could be found [9].

Measuring VPTs with MFV provided correlations to NCS that were comparable with those between NCS and NT, which is the current standard of VPT testing. Even though MFV and NT are methods that both assess VPTs, there are some notable differences between them. Firstly, NT is applied against the bone of either the medial malleolus or the big toe, while MFV is examined in the sole of the foot. An important difference between these sites is the distribution of cutaneous mechanoreceptors, where the Pacinian corpuscles are mainly found at the plantar side of the metatarsophalangeal joints [22]. Secondly, in comparison to NT, MFV assesses VPTs at multiple frequencies where each frequency is also automatically repeated four times. This makes it possible to recognize and reassess deviant measurement points, due to e.g., guessing or lack of concentration, which most likely contributes to a reduction of subjectiveness of the assessment. Thirdly, the NT is a hand-held device which, unlike the MFV, lacks internal control for contact force. This could lead to considerable investigator influence, given that VPTs are greatly affected by the contact force [23, 24]. These factors may have influenced the weaker correlations observed between NT and NCS, compared to those between MFV and NCS.

The results obtained from the ROC analysis indicated that a combination of only four frequencies (4, 8, 125 and 250 Hz) could be used instead of the full test of seven frequencies. Surprisingly, the AUC value for this reduced test was not only comparable, but in fact even slightly greater than for the full test. An AUC value of 0.83 indicates a good test but one should be aware of that the confidence interval of 0.73 to 0.93 indicates test values in a range between fair and excellent [25, 26]. Nevertheless, this finding provides a strong rationale for reducing the test procedure. Furthermore, the optimal cut-off value yielding the best result was a total Z-score of 0.5. This can be interpreted in two ways: either as a deviation of 0.5 standard deviations for each frequency, or as a deviation with e.g., two standard deviations for a single frequency. The latter interpretation aligns with the manufacturer’s guidelines for the VibroSense Meter®, whereas the suggested levels for total Z score are 1.7 for a suspected impairment and Z > 2.0 for a significant impairment [27]. However, it is important to note that our study population consisted of individuals with type 1 diabetes mellitus, and the comparisons were made between those with and those without DPN.

Since therapies for DPN is lacking, treatment is focused on prevention. Thus, identification of modifiable risk factors as well as methods for detection of early changes are key factors in such a strategy. Given the possibility of shortening the procedure, MFV testing could be performed in only 5–10 minutes, compared to NCS where a lower limb examination takes approximately 45 minutes. The selected combination of frequencies, consisting of both low and high frequencies (4, 8, 125 and 250 Hz), provides a broad picture and mirrors function of both Meissner and Pacinian corpuscles and their afferents. Impairment within low frequencies has been associated to risk of developing foot ulcers, whereas the higher frequencies seem to be affected both in early stages of diabetes as well as in the hand-arm vibration syndrome following use of vibrating hand-held tools [10, 13]. In addition to this, MFV has another important advantage of not being invasive and associated with the discomfort that some patients experience during NCS assessment. Lastly, previous work has shown that testing of VPTs can be performed repeatedly without any practice effects [28]. Hence, the test approach could be useful both in a clinical practice as well as in further research.

### Limitations

The study has some limitations. Originally, a larger participant sample was planned, however, the inclusion process was interrupted due to the COVID-19 pandemic and was not resumed. Despite our intention to include participants across a range from no DPN to severe DPN, locating individuals with slight impairments proved challenging. Consequently, our cohort consisted of very few patients with borderline conditions, which potentially impacted our results. Another limitation of the study is the exclusive inclusion of participants with type 1 diabetes mellitus. Because neuropathies seem to progress differently among diabetes subtypes [29], the findings may not be directly transferable to individuals with type 2 diabetes mellitus. This limitation also applies to other types of neuropathies.

## Conclusion

We found that there was a strong correlation between sural nerve amplitudes and VPTs measured with MFV at the 125 Hz frequency. We also showed that VPT testing based on two low and two high frequencies (4, 8, 125 and 250 Hz) has a higher classification efficacy for DPN than the full seven frequencies test. Testing could thus be shortened in time, in order to be more applicable in the clinical setting.
